# Supplementary material for: Live-cell imaging reveals enhancer-dependent Sox2 transcription in the absence of enhancer proximity
Source: eLife. 2019 May 24;8:e41769. doi: 10.7554/eLife.41769 (PMC6534382; doi:10.7554/eLife.41769)
Supplement: Supplementary file 1. — Protocols for targeting the cuO and/or tetO array(s) into genomic regions of interest in mouse ESCs. [file elife-41769-supp1.pdf]

OVERVIEW

Step 1: Landing site targeting

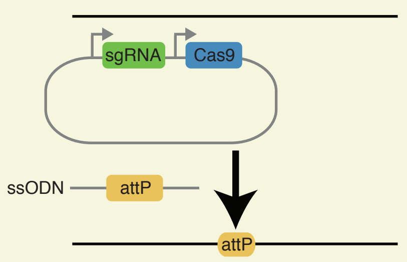

Summary

- Electroporate sgRNA/Cas9 expression plasmid and single-stranded oligodeoxynucleotide (ssODN)
- Dilute to clonal density after 72 hours
- Isolate colonies and screen by PCR

Step 2: Array plasmid integration

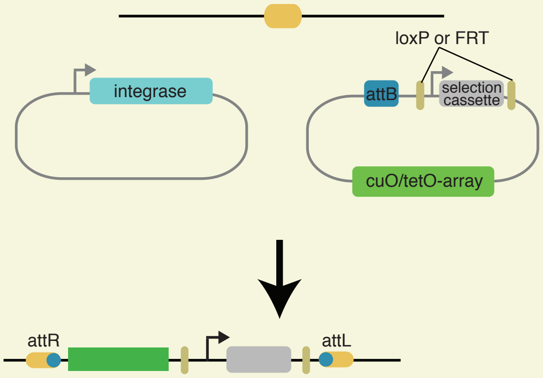

Summary

- Electroporate integrase expression plasmid and array targeting vector
- Drug selection or FACS sorting
- Dilute to clonal density
- Isolate colonies and screen by PCR

Optional Step 3: Selection cassette removal

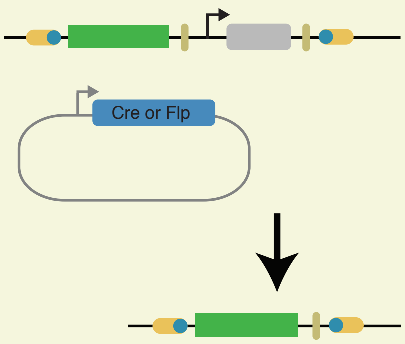

Summary

- Electroporate Cre or Flp expression plasmid
- Dilute to clonal density after 72 hours
- Isolate colonies and screen by PCR

attP ssODN Templates

XbaI — PhiC31 attP — XhoI

$(n)_{70}$tctagaccccaactggggtaacctttgagttctctcagttgggggctcgag$(n)_{70}$

5′ homology — 3′ homology

Bxb1 attP

$(n)_{65}$tctagagtcgtggtttgtctggtcaaccaccgcggtctcagtggtgtacggtacaaaccccgacctcgag$(n)_{65}$

Recombination Arms

PhiC31 attL
ggtgccagggcgtgcccttgagttctctcagttggggg

PhiC31 attR
ccccaactggggtaacctttgggctccccgggcgcg

Bxb1 attL
tcggccggcttgtcgacgacggcggtctcagtggtgtacggtacaaaccccgac

Bxb1 attR
gtcgtggtttgtctggtcaaccaccgcggtctccgtcgtcaggatcatccgggc

PCR Genotyping

Step 1

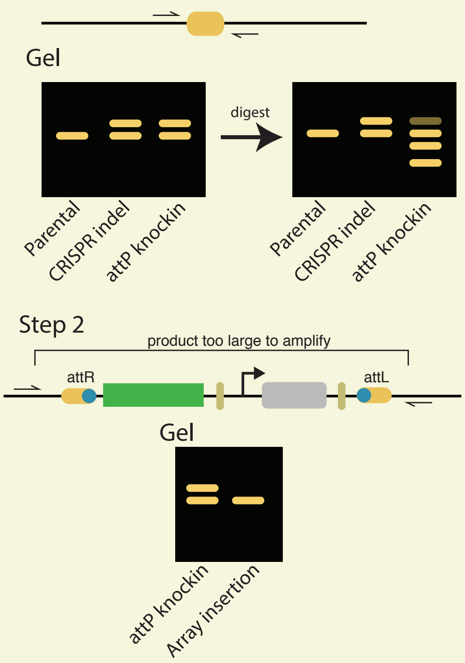

Protocols for Insertion of cuO-/tetO-arrays into Mouse ESCs

Reagents

0.1% gelatin -- Sigma ES-006-B
Accutase Solution -- Thermo Fisher MT25058CI
0.05% trypsin solution -- Thermo Fisher 25300-054
ES quality FBS -- Thermo Fisher SH3007002E
Neon Electroporation Kit -- Thermo Fisher MPK1096
GoTaq PCR Mix -- Promega M7123
D-PBS w/o Ca^{++} and Mg^{++} -- Thermo Fisher 14190-144
DMEM/F12 -- Thermo Fisher 11320-033
Neurobasal -- Thermo Fisher 21103-049
N2 Supplement -- Thermo Fisher 17502-048
B27 w/ Retinoic Acid -- Thermo Fisher 17504-044
7.5% BSA -- Thermo Fisher 15260-037
100X GlutaMax -- Thermo Fisher 35050-061
1-thioglycerol – Sigma M6145
PD03259010 – Selleckchem 1036
CHIR99021 – Selleckchem S2924
LIF – Peprotech 250-02

Recipes

2i media -- 50% DMEM/F12:50% Neurobasal, N2 Supplement, B27 Supplement, 0.05% BSA, 2 mM GlutaMax, 150 uM 1-thioglycerol, 1 uM PD03259010, 3 uM CHIR99021, 10^6 U/L LIF

Washing Media -- DMEM, 0.05% BSA

Oligonucleotide attP Homology Template Design

Design and order an oligonucleotide from IDT with the PhiC31 or Bxb1 attP sequence, flanking restriction sites for genotyping (XbaI and XhoI are recommended), and 65-70 bases of homology to the targeted genomic region of interest on each side of the attP insert (for a total of 200 bp). This can be ordered from IDT as an Ultramer. Place the attP insert sequence such that it is located between the CRISPR protospacer recognized by the sgRNA and the protospacer adjacent motif (PAM). This will ensure the knock-in allele will not be re-edited by CRISPR/Cas9. For more details regarding designing CRISPR sgRNAs and generating CRISPR/sgRNA expression vectors from pX330, see http://www.addgene.org/crispr/zhang/.

sgRNA | XbaI | PhiC31 attP | XhoI | PAM

$(n)_{50}$nnnnnnnnnnnnnnnnnnnntctagaccccaactggggtaacctttgagttctctcagttgggggctcgagnggnnnnnnn$(n)_{60}$

5′ homology arm (~70 bp) | 3′ homology arm (~70 bp)

sgRNA | XbaI | Bxb1 attP | XhoI | PAM

$(n)_{45}$nnnnnnnnnnnnnnnnnnnntctagagtcgtggtttgtctggtcaaccaccgcggtctcagtggtgtacggtacaaaccccgacctcgagnggnnnnnnn$(n)_{55}$

5′ homology arm (~65 bp) | 3′ homology arm (~65 bp)

Landing Site Targeting

Electroporation

1_Culture mouse ES cells for at least one passage subsequent to a thaw.
2_Add 0.5 mL 0.1% gelatin to 24 well plate for desired number of electroporation conditions. Incubate for 30 minutes.
3_Remove 0.1% gelatin and add 0.5 mL 2i media without antibiotics. Place in 37°C tissue culture incubator.
4_Dissociate ES cells with Accutase Solution for roughly 2 minutes at 37°C.
5_Transfer cell suspension to 15 mL conical and bring volume up to 10 mL with Washing Media.
6_Centrifuge at 300xg for 3 minutes at room temperature.
7_Wash cell pellet once with 10 mL Washing Media.
8_Centrifuge at 300xg for 3 minutes at room temperature.
9_Resuspend cells at approximately 10^6 cells/mL in Washing Media. Perform cell count.
10_Transfer 1.5 x 10^5 cells per electroporation condition to a fresh 15 mL conical.

___ # conditions x 150k cells = ___ total # of cells needed

11_Bring up to 10 mL in Washing Media.
12_Centrifuge at 300xg for 3 minutes at room temperature.
13_Wash once with 10 mL D-PBS w/o Ca^{++} and Mg^{++}.
14_Centrifuge at 300xg for 3 minutes at room temperature.
15_Carefully remove all traces of supernatant.
16_Resuspend cell pellet in R solution (see Neon Electroporation kit) at 10^7 cells/mL.

This works out to be 15 uL R solution for each 150,000 cells (i.e. each well of a 24-well plate). At this point, the protocol should be streamlined and completed as quickly as possible.

17_Aliquot 15 uL of cell suspension into individual 1.5 mL tubes, one for each electroporation condition (i.e. each well of a 24-well plate).
18_Add DNA for electroporation to each 1.5 mL tube.

400 ng pX330-sgRNA plasmid (at ~500-1000 ng/uL)
1 uL of 10 uM attP ssODN homology template

19_Use 10 uL Neon pipette tip to electroporate cells according the manufacturer's protocol. After electroporation, immediately add cells to one well of 24-well plate with pre-warmed 2i media.

Neon pipette will aspirate 10 uL (100,000 cells) for electroporation. Each condition is prepared at 15 uL volume to ensure that no air bubbles are introduced into Neon pipette, which will inhibit electroporation.

Recommended electroporation conditions
1400 V, 10 ms pulse width, 3 pulses

20_Allow 72 hours for recovery after electroporation, then proceed to dilution to clonal density (listed below).

Array Plasmid Integration

Electroporation

1_Culture mouse ES cells harboring attP knock-in for at least one passage subsequent to a thaw.
Steps 2-17 (as listed above)
18_Add DNA for electroporation to each 1.5 mL tube.

PhiC31- or Bxb1-mediated Plasmid Integration
400 ng integrase expression plasmid (at ~500-1000 ng/uL)
400 ng attB donor plasmid with tetO/cuO array (at ~500-1000 ng/uL)

PhiC31/Bxb1-mediated Double Plasmid Integration
300 ng PhiC31 integrase expression plasmid (at ~500-1000 ng/uL)
300 ng Bxb1 integrase expression plasmid (at ~500-1000 ng/uL)
300 ng PhiC31 attB donor plasmid with tetO array (at ~500-1000 ng/uL)
300 ng Bxb1 attB donor plasmid with cuO array (at ~500-1000 ng/uL)

19_Use 10 uL Neon pipette tip to electroporate cells according the manufacturer's protocol. After electroporation, immediately add cells to one well of 24-well plate with pre-warmed 2i media.

Neon pipette will aspirate 10 uL (100,000 cells) for electroporation. Each condition is prepared at 15 uL volume to ensure that no air bubbles are introduced into Neon pipette, which will inhibit electroporation.

Recommended electroporation conditions
1400 V, 10 ms pulse width, 3 pulses

20_Allow 72 hours for recovery after electroporation.
21_Passage electroporated cells. If using drug selection, add selective drug(s) to 2i media without antibiotics.

Appropriate concentration of drug should be determined empirically for each batch. However, a sensical place to start is listed below.
G418 selection – 500 ug/mL final concentration
Puromycin – 1 ug/mL final concentration

22_If using drug selection plasmids, culture cells under selection for the time necessary to kill all non-expressing cells. If using fluorescent reporter plasmids, culture cells for 7 days to allow unintegrated plasmids to be lost.

Necessary time for selection should also be determined empirically by treating non-expressing ES cells. However, the durations we observed were the following:
G418 selection – 6-7 days
Puromycin – 2 days

23_After selection is complete, proceed to dilution to clonal density (listed below).

Selection Cassette Removal

Electroporation

1_Culture mouse ES cells with integrated cuO and/or tetO arrays for at least one passage subsequent to a thaw.
Steps 2-17 (as listed above)
18_Add DNA for electroporation to each 1.5 mL tube.

FRT cassette removal
400 ng Flp expression plasmid (at ~500-1000 ng/uL)

Floxed cassette removal
400 ng Cre expression plasmid (at ~500-1000 ng/uL)

Double removal of FRT and Floxed cassette
400 ng Flp expression plasmid (at ~500-1000 ng/uL)
400 ng Cre expression plasmid (at ~500-1000 ng/uL)

19_Use 10 uL Neon pipette tip to electroporate cells according the manufacturer's protocol. After electroporation, immediately add cells to one well of 24-well plate with pre-warmed 2i media.

Neon pipette will aspirate 10 uL (100,000 cells) for electroporation. Each condition is prepared at 15 uL volume to ensure that no air bubbles are introduced into Neon pipette, which will inhibit electroporation.

Recommended electroporation conditions
1400 V, 10 ms pulse width, 3 pulses

Dilution to Clonal Density

1_ Add 7 mL 0.1% gelatin to 10 cm dish for each condition to isolate clones. Incubate for 30 minutes.
2_Dissociate ES cells with Accutase Solution for roughly 2 minutes at 37°C.
3_Transfer cell suspension to 15 mL conical and bring volume up to 10 mL with Washing Media.
4_Centrifuge at 300xg for 3 minutes at room temperature.
6_Resuspend in 100 uL Washing Media. Perform cell count.
7_Seed the pre-gelatinized 10 cm dish with 8 mL 2i media with ~5000 cells.
8_Allow single cells to grow into large, round colonies, changing media every 2 days.

Single ES cells in 2i will take 5-7 days to grow into large colonies ideal for picking.

Isolation of ES Cell Colonies

1_Treat a 96-well plate with 50 uL 0.1% gelatin per well. Incubate for 30 minutes.
2_Remove gelatin. Add 25 uL 0.05% trypsin to each well.
3_On day of colony picking, transfer 10 cm dish to tissue culture microscope for colony picking. Wipe down microscope and place in tissue culture hood if possible.
4_While viewing under microscope, use pipette tip to gently dislodge attached colony and aspirate colony into pipet tip. Transfer to one 96-well with trypsin.

Many colonies may already be dislodged at this point and can be directly aspirated from the growth media.

5_Continue collecting colonies and fill the 96-well plate column-wise (adding clones to wells A1-H1, followed by A2-H2). Once the second column is complete, add 100 uL 2i + 5% ES-quality serum to first column to quench trypsinization. Pipet up and down to dissociate colonies.
6_Repeat step 5 for each column sequentially (i.e. add 2i + 5% ES-quality serum to second column after third column colonies are picked).
17_After all colonies are picked, place in incubator and grow until near confluent (~2-3 days).

Media should be changed to 100 uL 2i on the following day to remove any trace trypsin activity.

18_Split plate of clones into 2 replica 96-well plates. Use one replicate for extracting DNA to use for PCR screening. Maintain the second plate in culture or freeze until genotyping is complete.

For additional details on these steps, we recommend *Manipulating the Mouse Embryo: A Laboratory Manual*

PCR Screening

attP Knock-in

1_Design genomic PCR primers that flank the attP knock-in site and have no overlap with the oligonucleotide template designed above.

2_Set up PCR reaction using GoTaq PCR Mix

PCR Reaction (per sample)

H_2O	8.5 uL
2x GoTaq Mix	10 uL
10 uM Primer Mix	0.5 uL
Genomic DNA (10-25 ng/uL)	1 uL

Total	20 uL

1- 95°C -- 5 minutes
2- 95°C -- 30 seconds
3- Primer Tm -- 30 seconds
4- 72°C -- 45-60 seconds
Repeat steps 2-4 an additional 34 times
5 - 72°C -- 5 minutes
6 - Hold at 12°C

3_Run 10 uL product on agarose gel. Identify samples that have an upward shifted band that is consistent with attP insertion.

4_To the remaining 10 uL of PCR product from promising samples, add 10 units XhoI or XbaI restriction enzyme.

Ensure that the enzyme used is not expected to digest the PCR product from unmodified genomic DNA.

5_Incubate at 37°C for 2 hours.

6_Run digested PCR on agarose gel. Identify samples where upper band has been digested and verify restriction fragment size. These samples are attP insertion clones.

These clones can be further validated by sequencing.

Array Plasmid Integration

1_Use the same PCR primers used to identify attP knock-in clones.

2_Set up PCR reaction using GoTaq

PCR Reaction (per sample)

H2O	6.125 uL
2x GoTaq Mix	7.5 uL
10 uM Primer Mix	0.375 uL
Genomic DNA (10-25 ng/uL)	1 uL

Total	15 uL

1- 95°C -- 5 minutes
2- 95°C -- 30 seconds
3- Primer Tm -- 30 seconds
4- 72°C -- 45-60 seconds
Repeat steps 2-4 an additional 34 times
5 - 72°C -- 5 minutes
6 - Hold at 12°C

3_Run products on agarose gel. Identify samples where the upward shifted band from the parental attP knock-in line has disappeared due to failure to amplify large cuO or tetO array insertion.

These clones can be further validated by using primer pairs that span the attR and attL junctions to confirm correct integration.

References

Nagy, A., Gerstenstein, M., Vintersten, K., Behringer, R., (2003). Manipulating the Mouse Embryo: A Laboratory Manual (Cold Spring Harbor Laboratory Press).
